# Der Fachkräftemangel in der Gefäßchirurgie – eine gemeinsame Aufgabe

**DOI:** 10.1007/s00104-023-01900-2

**Published:** 2023-06-22

**Authors:** U. Barth, F. Meyer, Z. Halloul

**Affiliations:** 1 Arbeitsbereich Gefäßchirurgie, Klinik für Allgemein‑, Gefäß- und Viszeralchirurgie, Helios Klinik Jerichower Land, Burg, Deutschland; 2 Arbeitsbereich Gefäßchirurgie, Klinik für Allgemein‑, Viszeral‑, Gefäß- und Transplantationschirurgie, Universitätsklinikum Magdeburg A. ö. R., Leipziger Straße 44, 39120 Magdeburg, Deutschland

**Keywords:** GefäßchirurgInnen, Ersatzbedarf, Mehrbedarf, Altersstruktur, WeiterbildungsassistentInnen, Nachwuchsgewinnung, Vascular surgeons, Replacement requirement, Additional needs, Age structure, Training assistants, Recruitment

## Abstract

**Einleitung:**

Auch in der Gefäßchirurgie wird ein zunehmender Mangel an FachärztInnen und AusbildungsassistentInnen beklagt. Trotz einer kontinuierlich steigenden Anzahl an ÄrztInnen und Humanmedizinstudierenden in Deutschland in den vergangenen Jahren ist der Bedarf an FachärztInnen und AusbildungsassistentInnen in der Gefäßchirurgie anhaltend enorm hoch.

**Methoden:**

Berufspolitische Analyse aus ärztlich-gefäßchirurgischer Sicht unter Einbeziehung aktueller verfügbarer Statistiken, vor allem des Statistischen Bundesamtes, der Bundesärztekammer sowie Landesärztekammer Sachsen-Anhalt (SA), sowie selektiver Referenzen der aktuellen medizinisch-wissenschaftlichen Literatur mit epidemiologischem Themenbezug.

**Ergebnisse:**

Im Jahr 2022 stellten laut der Grunddaten des Statistischen Bundesamtes 200 gefäßchirurgische Fachabteilungen insgesamt 5706 Betten zur Versorgung. Bei den Ärztekammern waren im Jahr 2021 1574 ÄrztInnen mit der Gebiets- und Facharztbezeichnung Gefäßchirurgie registriert. Im folgenden Jahr war ein Zuwachs von 404 GefäßchirurgInnen zu verzeichnen. Die Anerkennung der Facharztbezeichnung für Gefäßchirurgie sank von 166 im Jahr 2018 auf 143 im Jahr 2021.

In SA gibt es 23 gefäßchirurgische Versorgungseinheiten. Bei der Ärztekammer SA gab es 2021 52 registrierte ÄrztInnen mit der Fachgebietsbezeichnung Gefäßchirurgie im stationären Bereich. Im Vergleich dazu waren bei der Ärztekammer Nordrhein 2021 362 registrierte ÄrztInnen mit Gebiets- und Facharztbezeichnung Gefäßchirurgie insgesamt und 292 im stationären Bereich tätig. Die altersstandardisierte Krankenhausinzidenz der peripheren arteriellen Verschlusskrankheit (pAVK) stieg in den Jahren 2005 bis 2016 in Deutschland von ca. 190 auf über 250 pro 100.000 Einwohner und pendelte sich auf diesem Niveau ein. Dies entsprach einer relativen Zunahme um 33 %. Im gleichen Beobachtungszeitraum verdoppelte sich die Anzahl an durchgeführten Prozeduren, vor allem durch stark zunehmende endovaskuläre Eingriffszahlen (ca. 140 % Zuwachs) und Eingriffe bei arterieller Embolie/Thrombose (ca. + 80 %). Ein Forschungsgutachten im Auftrag der Deutschen Krankenhausgesellschaft (DKG) aus dem Jahr 2010 prognostizierte einen Ersatzbedarf an ÄrztInnen bis 2019 von gut 108.000 ÄrztInnen und einen Mehrbedarf von knapp 31.000 ÄrztInnen. Während bis 2020 noch 14,6–27,2 % der im Jahr 2008 Beschäftigten in Rente gehen, werden bis zum Jahr 2030 zwischen 45,6 und 68,5 % altersbedingt ausscheiden.

**Zusammenfassung:**

Trotz der statistisch nachweisbaren Verbesserung der Personalsituation an FachärztInnen für Gefäßchirurgie im stationären und ambulanten Bereich in Deutschland ist von einem Nachwuchsproblem auszugehen. Um die Nachwuchsgewinnung zielgerichtet zu gestalten, ist zunächst die umfängliche Erfassung der Grunddaten der Personalsituation und Personalentwicklung im Bereich der WeiterbildungsassistentInnen in der Gefäßchirurgie erforderlich. Darüber hinaus sollte weiter an der Umsetzung der bereits vor Jahren empfohlenen Handlungsempfehlungen wissenschaftlicher Gutachten auf Landes- und Bundesebene gearbeitet werden.

## Abstract (en)

**Introduction:**

An increasing shortage of specialists and training assistants is also being lamented in vascular surgery. Despite a continuous increase in the number of physicians and medical students in Germany in recent years, the need for specialists and training assistants in vascular surgery is enormous in a sustained manner.

**Methods:**

Professional policy analysis from a medical vascular surgery perspective including currently available statistics, especially from the Federal Statistical Office, the Federal Medical Association, the Saxony-Anhalt (SA) State Medical Association and selective references from current medical scientific literature on epidemiological topics.

**Results:**

In 2022, according to the basic data of the Federal Statistical Office 200 vascular surgery departments provided a total of 5706 beds for care. In 2021, 1574 physicians with the regional and specialist title in vascular surgery were registered by the medical associations. In the following years, there was an increase of 404 vascular surgeons. The recognition of the specialist title for vascular surgery fell from 166 in 2018 to 143 in 2021. There are 23 vascular surgery care units in Saxony-Anhalt (SA). At the SA Medical Association, there were 52 registered doctors with the specialist title in vascular surgery in the inpatient sector in 2021. In comparison, at the North Rhine Medical Association in 2021 there were 362 registered doctors with regional and specialist titles in vascular surgery overall and 292 in the inpatient sector. The age-standardized hospital incidence of peripheral arterial occlusive disease (PAOD) rose from approximately 190 to over 250 per 100,000 inhabitants in Germany between 2005 and 2016 and plateaued at this level. This corresponded to a relative increase of 33%. During the same observational period, the number of procedures performed doubled, mainly due to a strong increase in the number of endovascular interventions (approximately 140% increase) and interventions for arterial embolism/thrombosis (approximately + 80%). A research report commissioned by the German Hospital Society (DKG) in 2010 predicted a replacement requirement for physicians of approximately 108,000 by 2019 and an additional requirement of almost 31,000 physicians. While 14.6–27.2% of those employed in 2008 will have retired by 2020, between 45.6% and 68.5% will retire by 2030.

**Summary:**

Despite the statistically verifiable improvement in the staffing situation of specialists in vascular surgery in the inpatient and outpatient sectors in Germany, it can be assumed that there is a problem in recruiting young specialists. In order to target the recruitment of junior staff, it is first necessary to comprehensively record basic data on the staff situation and staff development in the area of residents in vascular surgery. In addition, further work should be done on implementing the recommendations for action already put forward years ago by scientific reports at state and federal levels.

## Hintergrund

Auch in der Gefäßchirurgie wird ein zunehmender Mangel an FachärztInnen und AusbildungsassistentInnen trotz einer kontinuierlich steigenden Anzahl an ÄrztInnen und Medizinstudierenden in Deutschland beklagt. Durch die bestehenden Lücken ist die Arbeitsbelastung der in der Klinik tätigen KollegInnen bei ungebrochenem Anstieg der PatientInnen mit Gefäßerkrankungen als angespannt zu bezeichnen. Die Corona-Pandemie scheint die Lage noch weiter verschärft zu haben, da die GefäßpatientInnenklientel auf den Stationen zurzeit aus überwiegend PatientInnen mit einer kritischen Extremitätenischämie besteht, die ein hohes Maß an ärztlicher Diagnostik, Therapie und Zuwendung bedürfen. Im Folgenden soll nun eine Bestandsaufnahme der und über die ärztlichen KollegInnen in der Gefäßchirurgie in Deutschland sowie am Beispiel strukturell unterschiedlicher Bundesländer, deren Wertung und mögliche Konsequenzen auch im Hinblick auf die zu erwartenden gesundheitspolitischen Veränderungen erörtert und diskutiert werden.

## Methode

Es erfolgte eine berufspolitische Analyse aus ärztlich-gefäßchirurgischer Sicht unter Einbeziehung aktueller verfügbarer Statistiken, vor allem des Statistischen Bundesamtes, der Bundesärztekammer sowie Landesärztekammer Sachsen-Anhalt (SA), sowie selektiver Referenzen der aktuellen medizinisch-wissenschaftlichen Literatur mit epidemiologischem Themenbezug.

## Ergebnisse

### Prognose der ärztlichen Entwicklung in Deutschland

Ein Forschungsgutachten im Auftrag der Deutschen Krankenhausgesellschaft (DKG) aus dem Jahr 2010 prognostizierte einen Ersatzbedarf an ÄrztInnen bis 2019 von gut 108.000 ÄrztInnen und einen Mehrbedarf von knapp 31.000 ÄrztInnen. Der Zugang an neuen ÄrztInnen bestünde im Wesentlichen aus Studierenden bzw. AbsolventInnen des Humanmedizinstudiums. Mittelbar müsse der Ersatz- und Mehrbedarf an ÄrztInnen komplett über den Krankenhausbereich gedeckt werden, da die Neuzugänge hier im Wesentlichen ihre Weiterbildung absolvierten. Der ÄrztInnenmangel wäre am frühesten und drastischsten im Krankenhaus spürbar und würde zu einem verschärften Wettbewerb zwischen ambulanter und stationärer Versorgung um Fachkräfte führen [1].

In einem 2. Gutachten über den Fachkräftemangel im stationären und ambulanten Bereich bis zum Jahr 2030 hat PricewaterhouseCoopers (PwC; Dienstleister in der Beratung und Prüfung von Unternehmen der Gesundheitswirtschaft) in Zusammenarbeit mit dem Darmstädter Forschungsinstitut WifOR im stationären Bereich bei OrthopädInnen und ChirurgInnen ein Defizit im Jahr 2030 von 7200 Vollzeitkräften prognostiziert. Außerdem wurde errechnet, dass ChirurgInnen und OrthopädInnen sowohl absolut als auch relativ mit einem hohen Ersatzbedarf konfrontiert werden. Während bis 2020 noch 14,6–27,2 % der im Jahr 2008 Beschäftigten in Rente gehen, werden bis zum Jahr 2030 zwischen 45,6 und 68,5 % altersbedingt ausscheiden [2].

### Gefäßchirurgische Versorgungssituation in Deutschland

**Figure 1:**
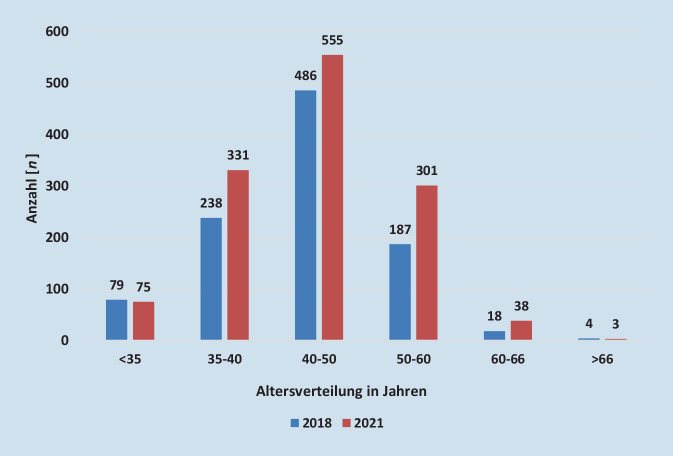

Im Jahr 2022 stellten laut der Grunddaten des Statistischen Bundesamtes 200 gefäßchirurgische Fachabteilungen insgesamt 5706 Betten zur Versorgung, was 6,9 Betten pro 100.000 Einwohner bei einem Nutzungsgrad von 63,5 % entspricht. Die Fallzahl betrug 174.151 [3]. Im Jahr 2021 gab es bei den Ärztekammern 1574 registrierte ÄrztInnen mit der Gebiets- und Facharztbezeichnung Gefäßchirurgie, im Jahr 2018 von 1170, was einem Zuwachs von 404 ÄrztInnen entspricht. Die Anerkennung der Facharztbezeichnung für Gefäßchirurgie sank von 166 im Jahr 2018 auf 143 im Jahr 2021 [4]. Im Jahr 2018 war die Altersgruppe von 40 bis 50 Jahren mit einem prozentualen Anteil von 48,02 % (*n* = 486) bei einer Gesamtzahl von 1012 GefäßchirurgInnen im stationären Bereich am häufigsten vertreten. Im Jahr 2021 betrug der Anteil dieser Altersgruppe nur noch 42,59 % (*n* = 555) bei einer Gesamtzahl von 1303, während der Anteil der GefäßchirurgInnen im Alter von 50 bis 60 Jahren von 18,5 % im Jahr 2018 auf 23,1 % im Jahr 2021 stieg ([3]; Abb. 1). Im Jahr 2018 kamen in Deutschland 1,41 GefäßchirurgInnen auf 100.000 Einwohner, dies steigerte sich bis zum Jahr 2021 auf 1,89 pro 100.000 Einwohner. Im ambulanten Tätigkeitsbereich waren es 2018 0,12 FachärztInnen pro 100.000 Einwohner, im Jahr 2021 0,24 pro 100.000 Einwohner [3].

Zur Verbesserung der Qualität der Versorgung gefäßchirurgischer PatientInnen wurde in den letzten Jahren die Bildung interdisziplinärer Gefäßzentren durch die Deutsche Gesellschaft für Gefäßchirurgie und Gefäßmedizin (DGG) in Kooperation mit der Privaten Akademie der DGG vorangetrieben. Im Februar 2023 waren hier 132 Gefäßzentren aufgelistet [5]. In Deutschland studieren aktuell rund 98.000 Personen das Fach Humanmedizin mit einem Frauenanteil von 62 %. Die Anzahl der StudienanfängerInnen hat in den vergangenen 10 Jahren leicht zugenommen. Im Jahr 2019 studierten rund 500 Personen mehr im 1. Fachsemester als im Jahr 2010. Die Anzahl der BewerberInnen für einen Medizinstudienplatz übertrifft die Anzahl der Studienplätze um ein Vielfaches [6].

### Gefäßchirurgische Versorgungssituation anhand eines strukturschwachen Bundeslandes – Sachsen-Anhalt

**Figure 2:**
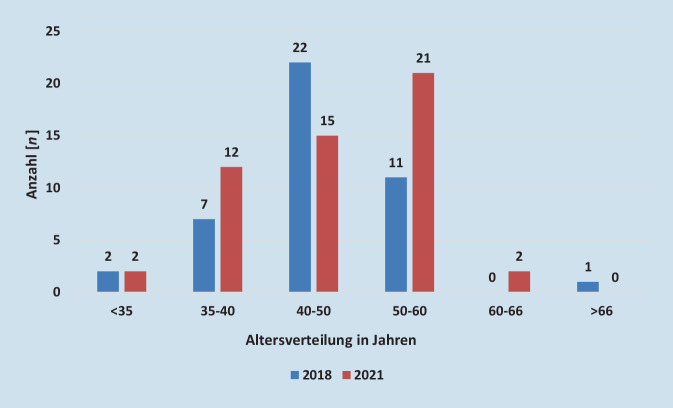

In SA gibt es 23 gefäßchirurgische Versorgungseinheiten, von denen 12 als integrativer Bestandteil einer Allgemein- und Viszeralchirurgie und 11 als eigenständige Klinik ausgewiesen sind. Einen universitären Lehrstuhl für Gefäßchirurgie gibt es an den zwei Universitätskliniken in SA nicht. Bei der Ärztekammer SA gab es 2021 52 registrierte ÄrztInnen mit der Fachgebietsbezeichnung Gefäßchirurgie im stationären Bereich, davon waren 2 KollegInnen im Alter von 60 bis 66 Jahren, 21 ÄrztInnen im Alter von 50 bis 60 Jahren, 15 GefäßchirurgInnen im Alter von 40 bis 50 Jahren, 12 im Alter von 35 bis 40 Jahren und 2 im Alter von unter 35 Jahren [3]. In der Altersgruppe 60 bis 66 Jahren kam es damit gegenüber 2018 zu einem Zuwachs von 2 ÄrztInnen, in der Altersgruppe 50 bis 60 von 10 KollegInnen und in der Altersgruppe zwischen 35 bis 40 von 5 GefäßchirurgInnen. Die Altersgruppe 40 bis 50 musste einen Rückgang um 7 ÄrztInnen verzeichnen (Abb. 2). Die GefäßchirurgInnenquote stieg von 1,99 pro 100.000 EinwohnerInnen auf 2,54 pro 100.000 EinwohnerInnen im stationären Bereich. Im ambulanten Tätigkeitsbereich waren 2018 0,05 FachärztInnen pro 100.000 EinwohnerInnen und 2021 0,14 pro 100.000 EinwohnerInnen registriert [3]. Nach Rücksprache mit der Ärztekammer SA im Dezember 2022 haben in den letzten 5 Jahren 28 ÄrztInnen die Facharztprüfung für Gefäßchirurgie bestanden. Die Anzahl der WeiterbildungsassistentInnen konnte nur auf Basis freiwilliger Angaben der Kammermitglieder mit 18 AssistentInnen ermittelt werden.

### Gefäßchirurgische Versorgungssituation anhand eines strukturstarken Bundeslandes – Nordrhein-Westfalen

**Figure 3:**
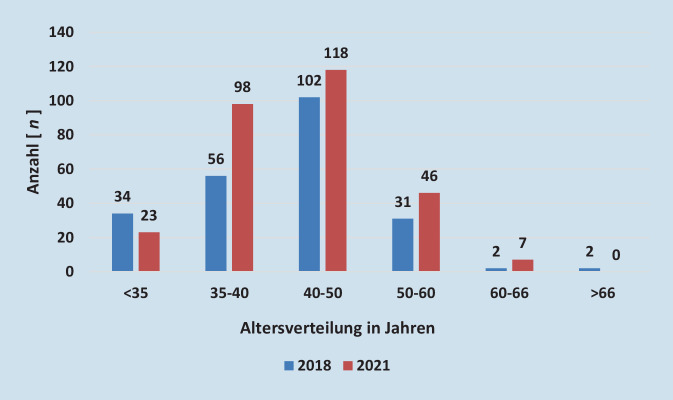

Im Vergleich zu SA sollen hier die Bedingungen der gefäßchirurgischen Situation in einem strukturstarken Bundesland (Nordrhein-Westfalen, NRW) genannt werden. Hier standen im Jahr 2020 1976 Betten in der Gefäßchirurgie zur Verfügung, was einer Quote von 11,0 Betten pro 100.000 Einwohner entsprach. Bei der Ärztekammer Nordrhein waren 2021 362 registrierte ÄrztInnen mit Gebiets- und Facharztbezeichnung Gefäßchirurgie insgesamt und 292 im stationären Bereich tätig. Davon waren 23 GefäßchirurgInnen unter 35 Jahren, 98 zwischen 35 und 40 Jahren, 118 zwischen 40 und 50 Jahren, 46 zwischen 50 und 60 Jahren und 7 zwischen 60 und 66 Jahren im stationären Bereich. In der Altersgruppe 35 bis 40 Jahren kam es damit gegenüber 2018 zu einem Zuwachs von 42 ÄrztInnen, in der Altersgruppe 40 bis 50 von 16 KollegInnen, in der Altersgruppe 50 bis 60 von 15 und in der Altersgruppe 60 bis 66 von 5 GefäßchirurgInnen. Im Jahr 2021 waren keine ÄrztInnen der Altersgruppe über 66 Jahren mehr registriert ([3]; Abb. 3).

### Entwicklung gefäßchirurgischer Erkrankungen in Deutschland am Beispiel der peripheren arteriellen Verschlusskrankheit

**Figure 4:**
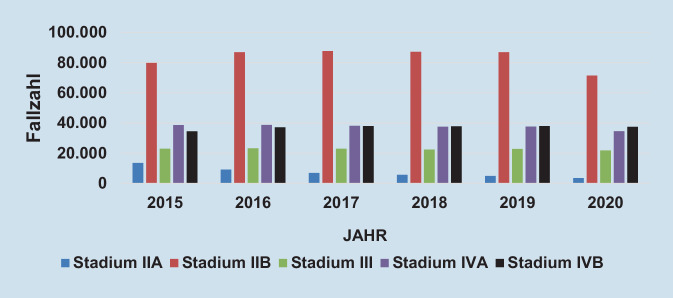

Die altersstandardisierte Krankenhausinzidenz der peripheren arteriellen Verschlusskrankheit (pAVK) stieg in den Jahren 2005 bis 2016 in Deutschland von ca. 190 auf über 250 pro 100.000 EinwohnerInnen und pendelte sich auf diesem Niveau ein. Dies entsprach einer relativen Zunahme um 33 %. Im gleichen Beobachtungszeitraum verdoppelte sich die Anzahl an durchgeführten Prozeduren, vor allem durch stark zunehmende endovaskuläre Eingriffszahlen (ca. 140 % Zuwachs) und Eingriffe bei arterieller Embolie/Thrombose (ca. + 80 %; [7]). Die Statistik der fallbezogenen DRG-Daten zeigt, dass insbesondere mit dem Auftreten der Corona-Pandemie die stationären Fallzahlen von PatientInnen mit einem pAVK-Stadium IIB bundesweit und in SA sanken. Die schweren pAVK-Stadien blieben in den Fallzahlen annähernd gleich, tendenziell in SA jedoch zunehmend (Abb. 4 und 5). Die altersstandardisierte Krankenhausinzidenz der Atherosklerose der Extremitätenarterien (I70.2) betrug im Jahr 2021 188,3 pro 100.000 EinwohnerInnen in Nordrhein-Westfalen und 220,5 pro 100.000 EinwohnerInnen in SA [3]. Dies bestätigt eine empfindliche Versorgungslücke von pAVK-PatientInnen in SA. Neben dem demographischen Wandel und der Pandemieproblematik scheint die ambulante Begleitung der pAVK-PatientInnen verbesserungswürdig zu sein. Rammos et al. zeigten in einer Studie, dass die Versorgung von pAVK-PatientInnen in Deutschland erschreckend mangelhaft ist. Nur 11 % der PatientInnen wurden im Jahr 2018 von einer/m GefäßchirurgIn und nur 8 % von einer/m AngiologIn behandelt. Nur die Hälfte der PatientInnen erhielt die leitliniengerechte Thrombozytenaggregations- und Statinmedikation [8]. Nach wie vor rangiert SA bei der Majoramputationsrate in Deutschland weit vorn. Bei einer Untersuchung der jährlichen bundesweiten Fallzahlen für die Jahre 2011 bis 2015 konnte gezeigt werden, dass überwiegend im Osten und Südosten höhere Amputationsraten bestehen. Insbesondere Kreise in Mecklenburg-Vorpommern, Brandenburg, Sachsen, SA, Thüringen und Bayern zeigten eine höhere „standardized mortality ratio“ (SMR) in mehreren Amputationshöhen. Diese auffälligen regionalen Unterschiede wurden durch die hohe altersadjustierte Prävalenz des Diabetes mellitus begründet [9]. Diese Situation scheint sich seit 2015 in den genannten Bundesländern nur marginal verbessert zu haben ([3]; Abb. 6). Dies bestärkt die Notwendigkeit einer Verbesserung der gefäßchirurgischen Versorgung in ländlichen und strukturschwachen Regionen. Bereits 2018 publizierten Udelnow et al. grundlegende Erkenntnisse der gefäßmedizinischen Versorgung in SA. Sie arbeiteten heraus, dass bereits vor 20 Jahren bevölkerungsbezogene Schätzungen zur für die Aufrechterhaltung der medizinischen Versorgung notwendigen Zahl niedergelassener SpezialistInnen publiziert worden waren. Hier wurde bereits eine Zahl von 1 AngiologIn auf 100.000 Einwohner gefordert. Die tatsächliche Zahl niedergelassener AngiologInnen und GefäßchirurgInnen in SA zusammen betrug zu diesem Zeitpunkt nur 0,87 auf 100.000 EinwohnerInnen für beide Fachgebiete. Aufgrund der demographischen Entwicklung ging man auch von einem weiter steigenden Bedarf aus [10].

**Figure 5:**
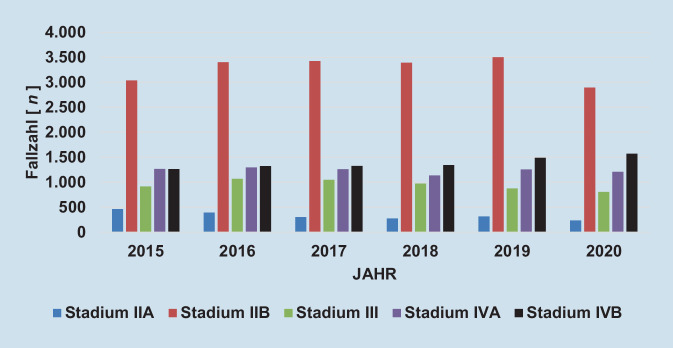

**Figure 6:**
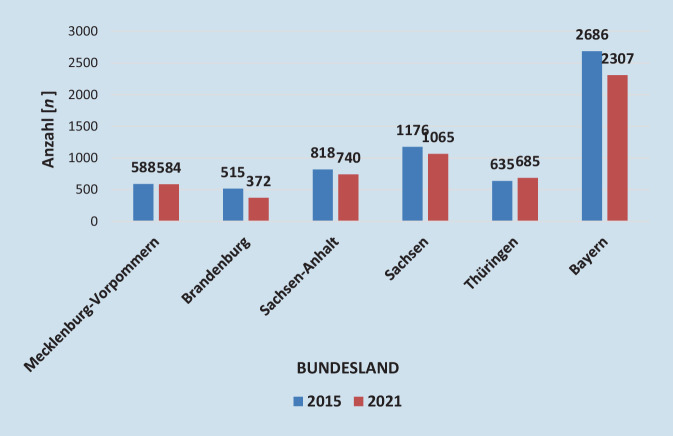

### Handlungsempfehlungen

Der Konvent leitender GefäßchirurgInnen Deutschlands, Österreichs und der Schweiz im Jahr 2007 und 2008 erkannte bereits die Herausforderungen der kommenden Jahre und warb um mehr Eigenständigkeit der Gefäßchirurgie i. S. einer unbedingten Klinikerhaltung bei Neuberufung eines universitären Klinikdirektors als auch Neuetablierung gefäßchirurgischer Universitätskliniken, mehr Präsentation und Repräsentanz der VertreterInnen der Gefäßchirurgie, Aufbau von Forschungskooperationen und -netzwerken, systematische Ausbildung gefäßchirurgischer Fachkräfte, höhere Transparenz wissenschaftlicher Aktivitäten und eine fachgesellschaftsübergreifende Zusammenarbeit [11].

In den vorgestellten Gutachten im Auftrag der DKG und dem Gutachten von PricewaterhouseCoopers in Zusammenarbeit mit dem Darmstädter Forschungsinstitut WifOR wird eine Reihe von Handlungsempfehlungen aufgezeigt, die im Folgenden erläutert werden.

1. Übereinstimmend empfehlen die beiden Gutachten eine Entlastung von Verwaltungsaufgaben und Dokumentationspflichten. Zu Recht wird der Dokumentationsaufwand im Krankenhaus als Ergebnis externer Dokumentationsanforderungen vor allem der Politik, der Selbstverwaltung sowie der KostenträgerInnen und des Medizinischen Dienstes der Krankenkassen (MDK) gewertet [1]. Durch Einstellung von Verwaltungskräften und den intelligenten Einsatz moderner IT-Systeme kann die Fokussierung ärztlicher Fachkräfte auf ihre angestammten Tätigkeiten erfolgen und sich die Attraktivität des Berufes deutlich erhöhen [2].
2. Professionelle Personalplanung und Personalentwicklung fördern die Anreize für eine befriedigende Tätigkeit und das Verbleiben im Unternehmen. Neben flexiblen Arbeitszeitmodellen kann ein auf MitarbeiterInnen bezogenes Konzept zur Gesundheitsförderung und Gesundheitsvorsorge bei der jungen Generation punkten [12]. Instrumente wie strukturierte oder standardisierte Einarbeitungs‑, Fort- und Weiterbildungskonzepte, Karriereplanungen, Beurteilungssysteme für Vorgesetzte und MitarbeiterInnen oder schriftliche Grundsätze der Mitarbeiterführung existieren bereits und können damit unproblematisch und aufwandsarm herangezogen sowie angewendet werden [1].
3. Als weiteres Mittel wird die Senkung der „Drop-out“-Raten im Medizinstudium und Erhöhung der Studienkapazitäten in der Humanmedizin erwähnt [1]. Die Anzahl der StudienanfängerInnen hat zwar in den letzten 10 Jahren zugenommen, aber ein Zuwachs von 500 StudienanfängerInnen im Jahr 2019 gegenüber dem Jahr 2010 [6] kann den kommenden Ersatz- und Mehrbedarf kaum decken.
4. Bei einem Frauenanteil von 62 % der Personen im Medizinstudium [6] sind familienorientierte Maßnahmen ein wichtiger Bestandteil zukünftiger Aufgaben im Gesundheitssystem. Besonders die Schaffung einer bedarfsgerechten und idealerweise betrieblichen Kinderbetreuung wird die Wahl des beruflichen Standortes der Ärztinnen bzw. jungen ÄrztInnenfamilien maßgeblich mit beeinflussen.
5. Die parallel entstandene Doppelversorgung durch strikte Trennung von ambulanter und stationärer Behandlung wird durch den entstehenden ÄrztInnenmangel ebenfalls infrage gestellt. Eine weitgehende und regelhafte Öffnung der Krankenhäuser für die ambulante Versorgung durch die Möglichkeit zur ambulanten fachärztlichen Behandlung durch KrankenhausärztInnen über persönliche und Institutsermächtigungen hinaus kann Versorgungslücken in der vertragsärztlichen Versorgung schließen [1, 2].
6. Bereits lange in der Diskussion ist die Neuordnung ärztlicher Aufgaben durch Delegation. Im Bereich von Dokumentation, Administration und Organisation können die ÄrztInnen entlastet werden [1]. Die Rahmenbedingungen für die Ausbildung und Etablierung von „physician assistants“ wurde durch die Kassenärztliche Bundesvereinigung (KBV) bereits 2017 definiert [13]. In der Gefäßchirurgie wurde ein strukturiertes Aus- und Weiterbildungsprogramm entwickelt und die neue Fachqualifikation „Gefäßassistent/In DGG®“ definiert und etabliert.
7. Ohne eine gezielt geförderte Zuwanderung kann der Mangel an ÄrztInnen nicht behoben werden, da der Wettbewerb um ÄrztInnen auch zunehmend international ausgetragen wird [2].

## Aktuelle Initiativen

Die DGG hat durch zwei Kampagnen die Initiative ergriffen. Die Kampagnen „Gefäßchirurgie macht Schule“ und „Tür auf“ sollen sich einerseits bereits SchülerInnen in der Oberstufe und Studierende für die Gefäßchirurgie interessieren. Umfangreiches Informationsmaterial und Unterlagen sind hier über die Internetseite der DGG abrufbar. Der Bund Deutscher Chirurgen (BDC) möchte mit seinem Projekt „Nur Mut! Kein Durchschnittsjob: ChirurgIn“ für das Fach Chirurgie begeistern sowie ganzheitlich informieren und unterstützen, eine bereits über Jahre aktive Initiative. Die Politik konzentriert sich im Wesentlichen auf lokaler Ebene um die Nachwuchsförderung im Hausärztebereich, meist durch gemeinsame Unterstützungsprojekte mit der KBV und den Krankenkassen. Die Ärztekammer SA möchte mit der Initiative „Arzt in Sachsen-Anhalt“ zentrale Informationen und Fördermöglichkeiten rund um eine ambulante und stationäre ÄrztInnentätigkeit in SA bündeln.

## Diskussion

Die personelle Situation hat sich, statistisch gesehen, sowohl in der Anzahl registrierter GefäßchirurgInnen im stationären Bereich als auch in der Quote pro 100.000 EinwohnerInnen im stationären und ambulanten Bereich bei zumindest statistisch nachweisbarem konstantem PatientInnenaufkommen in Deutschland bezüglich der pAVK verbessert. Auffällig ist eine Altersverschiebung der GefäßchirurgInnen überwiegend zu der Altersgruppe zwischen 50 und 60 Lebensjahren und den über 60-jährigen KollegInnen in Deutschland und insbesondere in SA. In NRW ist der Anstieg in der Altersgruppe zwischen 35 und 40 Jahren am stärksten. Neben der deutlich geringeren Anzahl an GefäßchirurgInnen in SA ist hier von einem höheren Altersdurchschnitt im Vergleich zu NRW auszugehen. Während hier nur 15,75 % der stationär tätigen GefäßchirurgInnen im Alter zwischen 50 und 60 Jahren sind, sind es in SA 40,38 %. Allein anhand dieses Unterschiedes lässt sich erkennen, dass der Nachwuchsmangel in SA früher zum Tragen kommt. Die Anzahl der AusbildungsassistentInnen in der Gefäßchirurgie in Deutschland und in den Bundesländern wird nicht erfasst und ist auf Basis der freiwilligen Angaben bei den Landesärztekammern nur schätzbar. Insofern kämen in SA bei 23 gefäßchirurgischen Versorgungseinheiten und 18 geschätzten AusbildungsassistentInnen 0,78 AssistentInnen auf eine gefäßchirurgische Einheit. Eine genaue statistische Nachweisführung eines Nachwuchsmangels in der Gefäßchirurgie ist aus den aktuell zugänglichen Daten nicht möglich. Die genaue Erfassung der Anzahl und des Ausbildungsstandes von gefäßchirurgischen AusbildungsassistentInnen ist eine dringliche Aufgabe, um eine verlässliche Bedarfsplanung zu generieren und gezielt Initiativen zur Rekrutierung zu führen. Auch wenn der aktuelle Stand des gefäßchirurgischen Nachwuchses statistisch nicht abgebildet werden kann, werden doch einige geplante gesundheitspolitische Entwicklungen der nächsten Jahre die Attraktivität der gefäßchirurgischen Weiterbildung beeinträchtigen. Die Empfehlungen des Instituts für Gesundheits- und Sozialforschung nach § 115b Abs. 1a SGB V umfasst die zusätzliche Aufnahme von 24 OPS-Codes im AOP-Katalog zur (perkutan-)transluminalen Implantation nichtmedikamentenfreisetzender, medikamentenfreisetzender, bioresorbierbarer und gecoverter Stents verschiedener Körperregionen, beschränkt auf einen Stent im gefäßchirurgischen Bereich [14]. Jedoch können nur ambulant tätige interventionelle RadiologInnen eine ambulante Intervention durchführen, was bedeutet, dass die Ausbildung der gefäßchirurgischen WeiterbildungsassistentInnen in endovaskulären Techniken für Einrichtungen mit einer endovaskulär gefäßchirurgischen Dominanz zunehmend schwieriger bis kaum überwindbar wird. Aktuell erfolgt die systematische Prüfung der gefäßchirurgischen Kliniken und Abteilungen durch den MDK zur Qualitätssicherungsrichtlinie zum Bauchaortenaneurysma (QBAA-RL). Es ist zu erwarten, dass einige gefäßchirurgische Einheiten im Zuge des durch die Corona-Pandemie verschärften Fachkräftemangels gerade in der intensivmedizinischen Fachpflege die geforderten Richtlinien nicht mehr umfänglich erfüllen und damit nicht mehr an der Versorgung von Bauchaortenaneurysmen teilnehmen können. Dies führt zu einem weiteren Ausbildungsdefizit, was die Attraktivität der gefäßchirurgischen Weiterbildung gerade in strukturschwachen Regionen weiter mildern sollte. Auch die geplante Änderung der Krankenhausstruktur durch Abstufung in 3 Krankenhauslevel könnte die bisher bestehende Attraktivität kleinerer gefäßchirurgischer Einheiten weiter schmälern und zur Abwanderung von AusbildungsassistentInnen in andere Fachgebiete fördern. Dies würde gerade in ländlichen Regionen wie SA die breite und qualitativ hochwertige gefäßchirurgische Versorgung gefährden. Inwieweit die Überwindung von Sektorengrenzen (stationär/ambulant), die Erweiterung des ambulanten Operierens und zunehmende Zentralisierung Einfluss auf die Personalsituation in der Gefäßchirurgie ausüben, kann nicht vorhergesagt werden. Bislang ist die Vergütung ambulanter gefäßchirurgischer Operationen, wobei hier nur Varizen und AV-Shuntoperationen verbleiben, da die Durchführung ambulanter (perkutan-)transluminaler Interventionen an eine(n) interventionell tätige(n) RadiologIn gekoppelt ist, unzureichend. Alternativ wäre hier das Konzept von operierenden niedergelassenen GefäßchirurgInnen zu nennen, wodurch sowohl Übergabesituationen zwischen den Sektoren und Fehler vermieden werden als auch gewährleistet wird, dass exakt die Therapie durchgeführt wird, die in langen Gesprächen und oft monatelangen abwägenden Prozessen mit PatientInnen sowie ggf. Angehörigen besprochen worden sind [15]. Somit würden ambulante Versorgungsdefizite ausgeglichen werden. Dies könnte für den Hauptanteil der GefäßchirurgInnen in Deutschland in der Altersgruppe zwischen 40 und 50 Jahren eine Alternative darstellen. Ungeachtet dessen, sollten vermehrte Anstrengungen zur Generierung des gefäßchirurgischen Nachwuchses in den nächsten Jahren erfolgen. Dazu werden zunehmend auch auf Lokal- und Länderebene Initiativen zur Gewinnung des Nachwuchses gestartet werden müssen. Die im Folgenden genannten Anregungen sollen als Diskussionsbasis für einen breiten Austausch verstanden werden und dazu anregen, weitere Lösungsvorschläge und Initiativen in die Diskussion mit einzubringen.

- Um die Bedeutung der Gefäßchirurgie zu unterstreichen, ist die Schaffung eines Lehrstuhls für Gefäßchirurgie (Gefäßmedizin) an den Universitäten dringend zu fordern, um die Gefäßchirurgie aus ihrem Sektionsdasein in klinischer und akademisch wissenschaftlicher Hinsicht herauszuholen. Die Gefäßchirurgie muss für Humanmedizinstudierende sichtbarer und präsenter werden [16]!
- Zudem sollten mehr landeseigene Weiterbildungen für junge AssistenzärztInnen angeboten werden. Zu denken ist dabei an Naht- und Interventionskurse, die in Zusammenarbeit mehrerer Kliniken organisiert und durchgeführt werden können, ohne eine Konkurrenzsituation zwischen den Kliniken zu erzeugen.
- Die Veränderung der Krankenhausstruktur erfordert die Schaffung neuer Ausbildungskooperationen zwischen Krankenhäusern mit unterschiedlichen Weiterbildungsermächtigungen, um die in der Weiterbildungsordnung verankerten Ausbildungsinhalte den WeiterbildungsassistentInnen vollständig anbieten zu können.
- Im Zuge dessen sollte entsprechend dem „Perspektivforum Junger Chirurgen“ der „Deutschen Gesellschaft für Chirurgie“ den gefäßchirurgischen WeiterbildungsassistentInnen eine Plattform auf Landesebene zum Austausch und breiter Diskussion gegeben werden.

## Schlussfolgerung

Trotz der statistisch nachweisbaren Verbesserung der Personalsituation an FachärztInnen für Gefäßchirurgie im stationären und ambulanten Bereich ist von einem Nachwuchsproblem auszugehen. Hier gibt es große regionale Unterschiede, wie am Vergleich zweier Bundesländer gezeigt. Um die Nachwuchsgewinnung zielgerichtet zu gestalten, ist zunächst die umfängliche Erfassung der Grunddaten der Personalsituation und Personalentwicklung im Bereich der WeiterbildungsassistentInnen in der Gefäßchirurgie erforderlich. Darüber hinaus sollte weiter an der Umsetzung der bereits vor Jahren empfohlenen Handlungsempfehlungen wissenschaftlicher Gutachten auf Landes- und Bundesebene gearbeitet werden. Eine Wende ist nur in Zusammenarbeit aller GefäßchirurgInnen im Land zu erreichen mit konkreten Vorschlägen und Forderungen an die Landespolitik und Klinikbetreiber. Leiteinrichtung und Koordinator sollten dabei die Universitätskliniken sein, die den unmittelbaren Zugang zu den Medizinstudierenden haben. Deshalb ist die Stärkung der Gefäßchirurgie durch Schaffung landeseigener Lehrstühle für Gefäßchirurgie dringend zu fordern.
